# Diffusion tensor imaging helps identify shunt-responsive normal pressure hydrocephalus patients among probable iNPH cohort

**DOI:** 10.1007/s10143-023-02078-1

**Published:** 2023-07-13

**Authors:** Vojtěch Sedlák, Adéla Bubeníková, Petr Skalický, Aleš Vlasák, Helen Whitley, David Netuka, Vladimír Beneš, Vladimír Beneš, Ondřej Bradáč

**Affiliations:** 1 Department of Radiology, Military University Hospital, Prague, Czech Republic; 2 Department of Neurosurgery and Neurooncology, 1st Faculty of Medicine, Charles University and Military University Hospital, Prague, Czech Republic; 3 Department of Neurosurgery, 2nd Faculty of Medicine, Charles University and Motol University Hospital, Prague, Czech Republic

**Keywords:** Diffusion tensor imaging, Normal pressure hydrocephalus, DTI, iNPH

## Abstract

The aim of this study was to investigate whether white matter changes as measured by diffusion tensor imaging (DTI) can help differentiate shunt-responsive idiopathic normal pressure hydrocephalus (iNPH) patients from patients with other causes of gait disturbances and/or cognitive decline with ventriculomegaly whose clinical symptoms do not improve significantly after cerebrospinal fluid derivation (non-iNPH). Between 2017 and 2022, 85 patients with probable iNPH underwent prospective preoperative magnetic resonance imaging (MRI) and comprehensive clinical workup. Patients with clinical symptoms of iNPH, positive result on lumbar infusion test, and gait improvement after 120-h lumbar drainage were diagnosed with iNPH and underwent shunt-placement surgery. Fractional anisotropy (FA) and mean diffusivity (MD) values for individual regions of interest were extracted from preoperative MRI, using the TBSS pipeline of FSL toolkit. These FA and MD values were then compared to results of clinical workup and established diagnosis of iNPH. An identical MRI protocol was performed on 13 age- and sex-matched healthy volunteers. Statistically significant differences in FA values of several white matter structures were found not only between iNPH patients and healthy controls but also between iNPH and non-iNPH patients. ROI that showed best diagnostic ability when differentiating iNPH among probable iNPH cohort was uncinate fasciculus, with AUC of 0.74 (*p* < 0.001). DTI methods of white matter analysis using standardised methods of ROI extraction can help in differentiation of iNPH patients not only from healthy patients but also from patients with other causes of gait disturbances with cognitive decline and ventriculomegaly.

## Introduction

Idiopathic normal pressure hydrocephalus (iNPH) is a clinical syndrome classically characterized by a triad of progressive dementia, gait disturbances, and urinary incontinence, with imaging findings of ventriculomegaly despite normal opening pressures on lumbar puncture [1]. The reported prevalence of iNPH in elderly individuals lies usually within 1–2% range [2], with some studies reporting up to 5.9% prevalence among individuals of 80 years of age and older [3]. Therefore, iNPH is a significant burden not only to the affected and their families but also to healthcare systems as a whole. Consequently, accurate and timely diagnosis of iNPH is crucial, probably even more so than in some other neurodegenerative disorders, as progression of many of the iNPH symptoms can be slowed down or even reversed by timely shunt-placement surgery [4, 5]. Several studies also demonstrated that shorter duration between symptom onset and shunt surgery yields better response to shunt placement and general outcome [6, 7] and that shunt placement is a cost-effective treatment method [8].

However, clinical features of iNPH are grossly non-specific as individual components of Hakim’s triad are ubiquitous in the elderly population and often coincide without an underlying iNPH^2^. iNPH is associated with certain imaging findings [9, 10] and a scoring system has been developed (iNPH Radscale), which seems to differentiate well between shunt-responsive iNPH patients and asymptomatic controls [11]. However, the discriminatory power between shunt responders and symptomatic nonresponders is relatively low [12–14]. Therefore, current clinical practice still relies on complex multi-modality evaluation often requiring inpatient-based workup. Therefore, given the prevalence, the search for novel iNPH biomarkers is ongoing, especially for those that could reliably differentiate shunt responders from non-responders among the probable iNPH group. One promising method that might non-invasively improve diagnosis of iNPH on an outpatient basis is diffusion tensor imaging (DTI) [15], a magnetic resonance imaging (MRI) technique based on characterizing movement of water molecules in tissues. In white matter, diffusion characteristics of water molecules are influenced by the microarchitecture of axonal fiber bundles; therefore, it is possible to assess white matter cohesion and integrity as well as principal orientations of its pathways [16].

Several studies [17, 18] investigated the ability of DTI to differentiate between iNPH and healthy controls, usually with statistically significant differences between cohorts. However, in clinical practice, differentiating between iNPH patients and healthy subjects is rarely an issue. A more daunting and clinically important task is to differentiate shunt-responsive iNPH patients from patients with other neurodegenerative disorders, especially those presenting with gait disturbances. Also, non-standardized methods of investigating white matter, e.g., not using clear definition of evaluated regions of interest (ROI) or the use of hand-drawn ROI, make reproduction of reported results difficult.

In order to further investigate the clinical utility of DTI in diagnosis of iNPH, this study focused on differentiation of shunt-responsive iNPH patients from non-responders among a cohort of probable iNPH patients. Intending to achieve better reproducibility, data was co-registered to a standard MNI152 space, regions of interest were extracted using JHU white matter atlas, and FSL’s TBSS pipeline was used for analysis of white matter skeleton.

## Materials and methods

This study was approved by the ethics board of the Military University Hospital Prague. Informed consent was obtained from all patients prior to the inclusion into the database and following procedures.

### Patient identification, clinical assessment

Patients referred to the Military University Hospital Prague between 2017 and 2022 with a working diagnosis of probable iNPH were prospectively included in the study. Criteria for inclusion in the study included (1) age above 40 years old, (2) insidious onset of symptoms over period of at least 3 months, (3) ventriculomegaly defined as Evans’ index > 0.3, (4) gait disturbance and at least one of the remaining Hakim’s triad symptoms, i.e., urinary incontinence and/or cognitive deficit, and (5) no other known underlying condition that would account for the symptoms (1). Gait disturbance was evaluated using the Dutch Gait Scale.

After admission, patients underwent a complex diagnostic protocol consisting of clinical, psychological, imaging, and CSF studies. Toward the end of these tests, all patients underwent a lumbar infusion test (LIT). Once the test was completed, lumbar drainage was performed using the same needle, CSF was drained for 120 h, and clinical evaluation was repeated. Ventriculoperitoneal shunt (VPS) implantation was indicated for all patients with (1) positive result on LIT (resistance to CSF outflow > 9 mmHg/ml/min) and (2) a minimum of 15% clinical improvement in the Dutch Gait Scale following the 120-h lumbar drainage (LD). If these criteria were not fulfilled, the patient was determined as non-iNPH. Patients with abnormal CSF opening pressure (> 20 cm H_2_O) and/or abnormal laboratory findings in CSF were excluded from the study.

A multimodal MRI protocol was performed on all included patients prior to CSF testing. In addition, 13 age- and sex-related healthy controls underwent the same imaging protocol. Subjects with secondary NPH, other apparent causes of hydrocephalus, and/or other major pathology on MRI examination were excluded from the study.

### MRI data acquisition

All patients were examined on a 3 T MRI system GE 750w (GE Healthcare, Chicago, Illinois) stationed at the Department of Radiology of The Military University Hospital Prague. Standardized imaging protocol consisted of 3D T1, 3D T2, and DTI sequences, accompanied by a phase-contrast CSF-flow study and fMRI acquisition. The DTI sequence covering the whole brain had the following parameters: FOV 256 mm, acquisition matrix 128 × 128, slice thickness 4 mm, TR 8000 s, TE 77 s, and bandwidth 1930 Hz. Monopolar diffusion scheme with 27 diffusion directions at *b*-value of 1000 s/mm^2^ and 3 b0 acquisitions was used. Total scanning time for the DTI sequence was 4 min, 8 s.

### MR data analysis

**Fig. 1:**
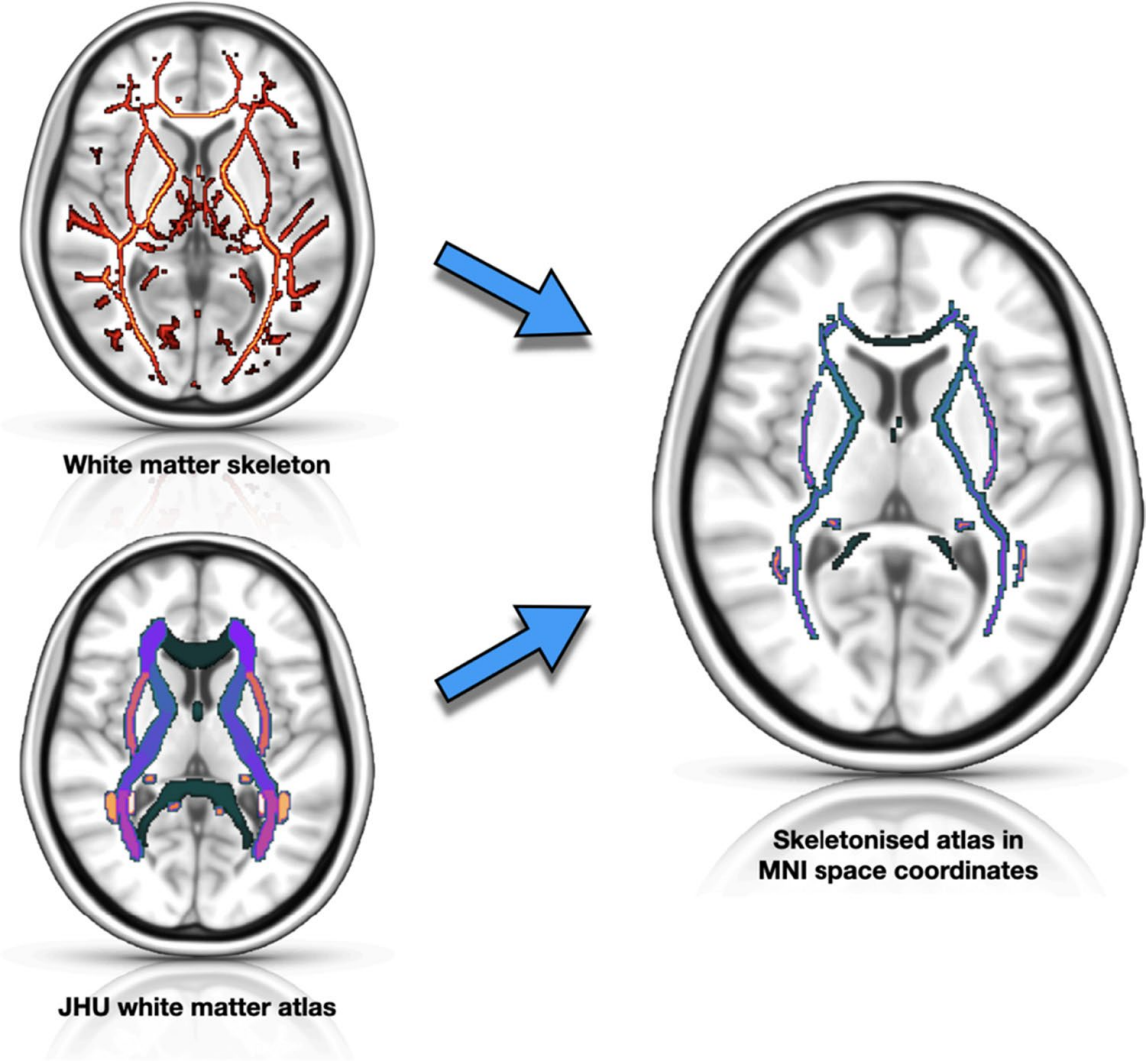
Creation of skeletonized white matter ROI using JHU atlas within MNI152 space

DTI data were processed using the FSL toolkit (FMRIB software library, University of Oxford, UK) [19, 20]. Preprocessing included eddy current distortion correction and N4 bias field correction. Preprocessed data then entered the tract-based spatial statistics (TBSS) [21] pipeline. First, fractional anisotropy (FA) images were created by fitting a tensor model to the raw diffusion data using FDT, and then brain extracted using BET [22]. All subjects’ FA data were then aligned into a common MNI152 space using the nonlinear registration tool FNIRT [23–25]. Next, the mean FA image was created and thinned to create a mean FA skeleton which represents the centers of all tracts common to the group. Each subject’s aligned FA data was then projected onto this skeleton. Individual ROIs were extracted from each patient’s white matter skeleton using JHU white-matter tractography atlas [26] (Fig. 1). Mean diffusivity (MD) values were extracted for each patient as well.

### Statistical analysis

Differences of categorical variables were standardly evaluated using the chi-square test. Comparisons of two continuous variables were calculated using *t*-tests for independent samples. Multiple intergroup comparisons were performed using ANOVA with subsequent Fisher LSD post hoc testing. Significant predictors were further implemented for their diagnostic accuracy evaluation by calculating sensitivity, specificity, receiver operating characteristic (ROC) curve, and the area under the curve (AUC). All calculations and graphical interpretations were performed in STATISTICA (TIBCO Software) and OriginPro Software (OriginLab Corporation).

## Results

During the 6-year period, a total of 173 patients with suspected iNPH were prospectively enrolled in the study. Patients with other discernible causes of their symptoms (e.g., evidence of previous stroke, neoplasms, other types of hydrocephalus) were excluded. Three patients refused shunt insertion. The final study cohort consisted of 85 participants with adequate data from DTI and CSF functional testing. Out of these 85, 47 patients were diagnosed with shunt-responsive iNPH and indicated for VPS surgery. The remaining 38 patients did not exhibit any significant improvement on LIT and/or LD tests and were considered to have either shunt non-responsive iNPH or another neurodegenerative disorder (collectively referred to as non-iNPH group in the following text for the sake of conciseness). Remaining 13 participants consisted of age- and sex-related healthy controls. Demographic data related to included patients are presented in Table 1.

**Table 1:** Demographic details of included study populations

	iNPH	Non-NPH	Controls	*p*-value
N. of patients (%)	47 (48.0)	38 (38.8)	13 (13.3)	
Male	32 (53.3)	21 (35.0)	7 (11.7)	0.407
Female	15 (39.5)	17 (44.7)	6 (15.8)	
Age in years, mean ± SD	73.1 ± 6.0	74.5 ± 7.6	68.2 ± 12.5	0.046
Gait score pre-ELD, mean ± SD	28.1 ± 9.1	24.6 ± 10.7	–	0.106
Gait score post-ELD, mean ± SD	23.3 ± 9.6	23.6 ± 10.9	–	0.905
MoCA, mean ± SD	18.4 ± 5.8	16.8 ± 5.2	–	0.292

### iNPH vs non-iNPH

**Fig. 2:**
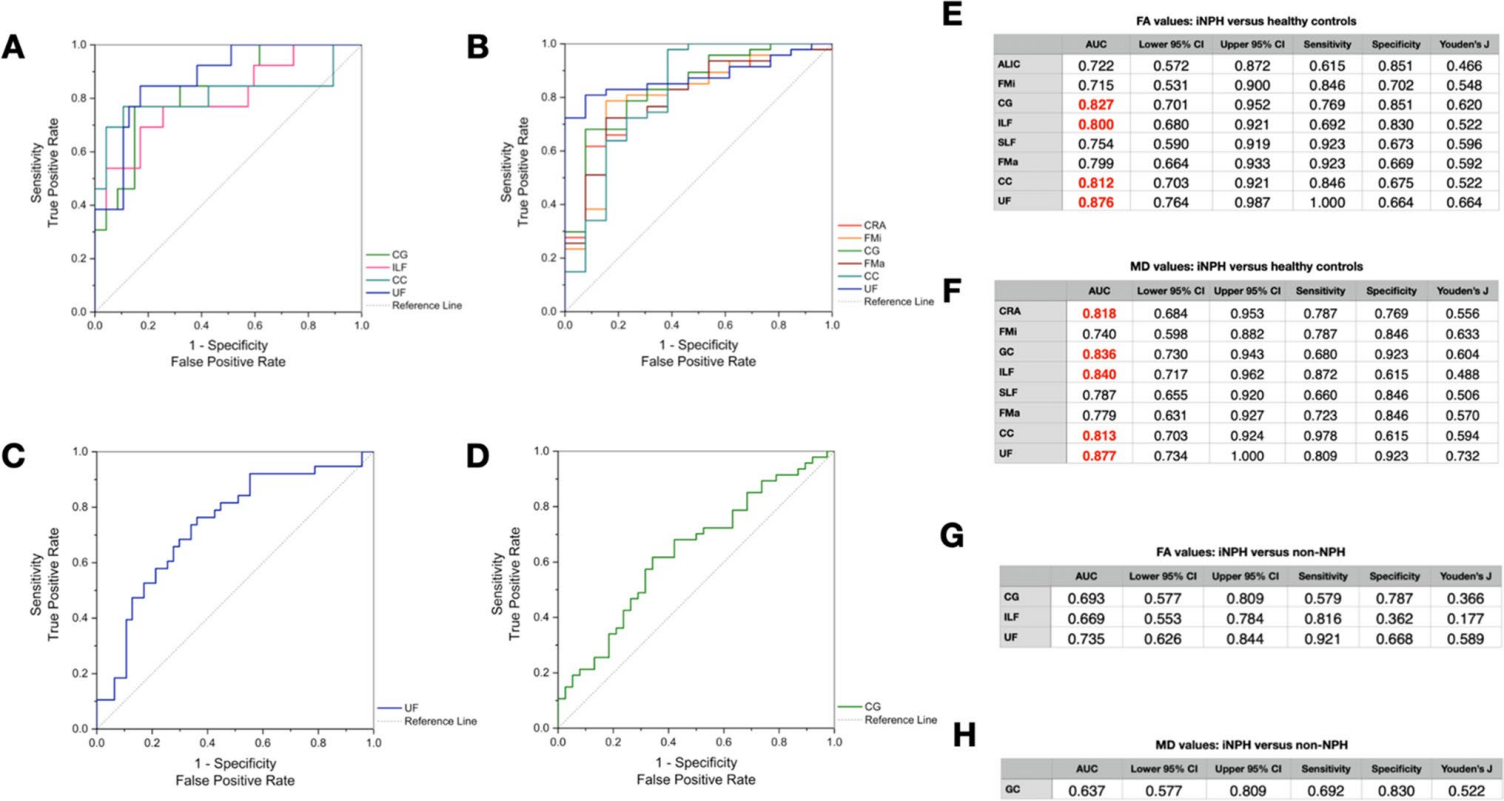
ROC curves for **A** FA and **B** MD values in discrimination of iNPH from healthy controls. In both ROC curves, depicted are only the predictors with AUC 0.80 (in tables presented in red bold). **E**, **F** All variables along with the remaining statistically significant predictors from the univariate analysis are presented in related tables below with calculated AUC, associated 95% CI, specificity, sensitivity, and Youden’s *J* index. Analogously, differences between iNPH and non-NPH are presented in graphs and tables C, D, G, and H

According to the predefined binary groups, i.e., (1) iNPH and healthy controls and (2) iNPH and non-NPH, individual diagnostic ability for each group was calculated. Overview of detailed ANOVA analysis regarding FA values of individual anatomical structures among NPH, non-NPH, and healthy controls is presented in Table 2. When differentiating between iNPH and non-NPH patients, ROI whose FA values showed the highest AUC were the uncinate fasciculus (AUC 0.74), cingulate gyrus (AUC 0.69), and the inferior longitudinal fasciculus (AUC 0.67). MD values of the cingulate gyrus showed AUC of 0.64. See Fig. 2 for the most distinctive FA and MD values between iNPH and non-NPH in individual structures.

**Table 2:** Summary of analyzed FA values within individual anatomical landmarks. Each value is presented as mean ± SD

	NPH	Non-NPH	Controls	*p*-value
CRA	0.367 ± 0.051	0.374 ± 0.047	0.393 ± 0.025	0.213
ALIC	0.494 ± 0.057	0.510 ± 0.054	0.534 ± 0.048	0.059
CP	0.635 ± 0.034	0.647 ± 0.030	0.638 ± 0.032	0.269
CRS	0.478 ± 0.053	0.469 ± 0.058	0.450 ± 0.041	0.249
FMi	0.584 ± 0.052	0.600 ± 0.045	0.621 ± 0.034	**0.037**
CG	0.378 ± 0.059	0.416 ± 0.044	0.454 ± 0.052	** < 0.001**
ILF	0.442 ± 0.044	0.450 ± 0.041	0.491 ± 0.043	**0.002**
PLIC	0.696 ± 0.032	0.702 ± 0.032	0.695 ± 0.020	0.605
RLIC	0.564 ± 0.050	0.571 ± 0.032	0.569 ± 0.033	0.798
SLF	0.383 ± 0.057	0.418 ± 0.050	0.429 ± 0.043	**0.003**
FMa	0.641 ± 0.090	0.677 ± 0.079	0.723 ± 0.074	**0.006**
CC	0.475 ± 0.045	0.493 ± 0.053	0.539 ± 0.064	** < 0.001**
UF	0.336 ± 0.068	0.394 ± 0.062	0.425 ± 0.041	** < 0.001**
CRP	0.413 ± 0.066	0.430 ± 0.049	0.448 ± 0.043	0.107

Significant values are presented in bold text

Used abbreviations: *CRA*, anterior corona radiata; *ALIC*, anterior limb of the internal capsule; *CP*, cerebral peduncle; *CRS*, superior corona radiata; *FMi*, forceps minor; *CG*, cingulate gyrus; *ILF*, inferior longitudinal fasciculus; *PLIC*, posterior limb of the internal capsule; *RLIC*, retrolenticular limb of the internal capsule; *SLF*, superior longitudinal fasciculus; *FMa*, forceps major; *CC*, body of corpus callosum; *UF*, uncinate fasciculus; *CRP*, posterior corona radiate

### iNPH vs healthy controls

By studying differences between iNPH and healthy controls, statistically significant differences in FA as well as MD were found in the majority of investigated white matter structures. ROI with best discriminating power were the uncinate fasciculus (AUC 0.88 and 0.88 for FA and MD values, respectively) and cingulate gyrus (AUC 0.83 and 0.84). Figure 2 depicts both models (FA and MD) with the most distinctive parameters (AUC ≥ 0.80).

## Discussion

### Study impact

In a clinical setting, iNPH is often a challenging condition to diagnose due to relatively non-specific imaging and clinical features, and currently there is a lack of non-invasive outpatient procedures that can reliably confirm the diagnosis or at least trim the cohort of probable iNPH patients to numbers that would be feasible for thorough inpatient evaluation.

This is one of the possible reasons for the focus on MRI in the iNPH-related scientific literature, as a variety of complex evaluations of brain morphology and function are achievable in the outpatient setting. Unfortunately, standard brain MR protocols lack sufficient sensitivity and/or specificity to be a confirmatory or screening test on its own, and therefore search for novel approaches to imaging diagnosis of iNPH is ongoing.

A common challenge for application of DTI in the clinical setting is the lack of standardized approaches to extraction of DTI measures. Results reported in this study were achieved using standardized approaches widely employed in the literature (TBSS pipeline), with alignment of FA and MD data into standard MNI152 space and ROI placement according to JHU white-matter atlas. Both MNI152 template and JHU white-matter atlas are accessible open-source references that allow for easy reproduction of the methodology.

### iNPH vs healthy controls

There is a decent amount of studies [17] showing the ability of DTI measures to detect changes in white matter of iNPH patients and differentiate them from healthy controls. Commonly reported structures with altered FA or MD values in iNPH include CC, FMi, ALIC, or the corticospinal tract (CST) [27]. Our study confirmed the findings for the first three of these regions, but due to the use of JHU atlas, CST was not evaluated as a single unit, but instead its parts were included in several other ROI (cerebral peduncle, PLIC, corona radiata), which might contribute to the less convincing association of iNPH and CST changes in our study. This study also found differences in several other areas of white matter (UF, FMa, ILF, SLF), which are less commonly mentioned in literature and arguably deserve more attention when investigating the pathogenesis of iNPH.

UF and CG are considered to be a part of the limbic pathway, and while the former is reported to play a role in certain types of learning (e.g., trial-and-error or reversal learning) and memory retrieval (e.g., name retrieval) [28], the former is involved in executive function [29]. Our study found a significant decrease in FA of these structures, which along with increased MD values in the CRA (possibly attributable to glial changes) might contribute to frontal symptomatology of iNPH patients [30]. On the other hand, visuospatial and visuoperceptual deficits of iNPH patients might be related to disruption of ILF [31], which in the iNPH group exhibited significantly lower FA values and higher MD values compared to healthy controls. Involvement of the above-mentioned limbic pathway structures could also help explain impaired attention of iNPH patients.

### iNPH vs non-iNPH

This study suggests that DTI can be a useful tool in iNPH workup, as it has the ability to discriminate not only between iNPH patients and healthy controls but also between iNPH patients and other causes of gait disturbances and cognitive decline. DTI measures of several structures showed high diagnostic ability in differentiating iNPH and non-iNPH, like, e.g., FA values of the UF, which showed sensitivity of 92% and specificity of 67% (AUC of 0.735), which is more than what is usually achievable by MRI evaluation solely based on morphological features [11–14]. Other structures that might be useful in distinguishing iNPH from non-iNPH were the CG, CC, and ILF. These findings are mostly in-line with available literature; however, available articles focusing on DTI-based differences between iNPH patients and other neurodegenerative disorders are few and far in between and usually include only a limited number of patients [18].

## Limitations of this study

A common limitation in the research of neurodegenerative disorders is the decreased compliance of these patients, older age, and presence of other conditions often precluding (e.g., pacemaker implant) or confounding (e.g., concomitant neurodegenerative disorder) certain diagnostic or therapeutic approaches. In the case of our study, this meant excluding patients with insufficient quality of the imaging data or another pathology found on MRI exam (i.e., tumor, post-ischemic changes). In this context, however, DTI is a technique relatively resilient to motion artifacts, with possible motion-correction during post-processing, which makes it a suitable method for low-compliance patients.

An important thing to consider is also how the diagnosis of iNPH was reached. At our institute, the diagnosis of iNPH was made when (1) the clinical criteria for probable iNPH were met along with (2) clinical improvement after 120-h lumbar drainage (measured as 15% improvement on the Dutch gait scale) and when (3) no other underlying cause explaining patient symptoms was found. As these criteria were set as relatively strict, it is possible that some patients harboring iNPH in its initial phase were tested as false negatives. A 120-h lumbar drainage was used to maintain diagnostic uniformity and at the same time to compare DTI with the gold standard of iNPH functional testing. Some patients may also improve clinically during cerebrospinal fluid diversion with delay [32]. Additionally, due to extended lumbar drainage, data of testing of some patients were high of artefacts (i.e., periprocedural movements, speaking, coughing) and thus the data were not used in the study analysis.

## Conclusion

iNPH is a challenging condition to correctly diagnose and manage, with novel iNPH-specific biomarkers being in high demand. This study focused on the application of DTI in differentiation of shunt-responsive iNPH patients from other causes of gait disturbances and cognitive decline, refractory to CSF derivation. When using ROIs extracted from standardized JHU white-matter atlas, DTI measures of several white matter regions showed promising results, reaching 92% sensitivity and 67% specificity when differentiating shunt-responsive iNPH patients from patients who fulfilled criteria of probable iNPH but did not improve on CSF derivation. We find these results encouraging and warranting further search for iNPH-specific diffusion MRI biomarkers.

## Data Availability

All data and materials are available from the corresponding author upon a reasonable request.
